# Supplementary material for: Assessing the sustainability of yellow anaconda (Eunectes notaeus) harvest
Source: PLoS One. 2023 Jan 12;18(1):e0277629. doi: 10.1371/journal.pone.0277629 (PMC9836266; doi:10.1371/journal.pone.0277629)
Supplement: S1 File — (DOCX) [file pone.0277629.s001.docx]

**Supplementary Information**

**Assessing the sustainability of yellow anaconda (*Eunectes notaeus*) harvest**

Bruno F. Camera, Itxaso Quintana, Christine Strüssmann, Tomás Waller, Mariano Barros, Juan Draque, Patrício A. Micucci, Everton B. P. Miranda*

B. F. C., C. S., I. Q., and E. B. P. de M. conceived and designed the experiments, and wrote the paper. T. W., M. B., J. D., and P. A. M. provided data, revised, and made improvements to the paper.

*Contact information: Everton Miranda

E-mail address: mirandaebp@gmail.com

**
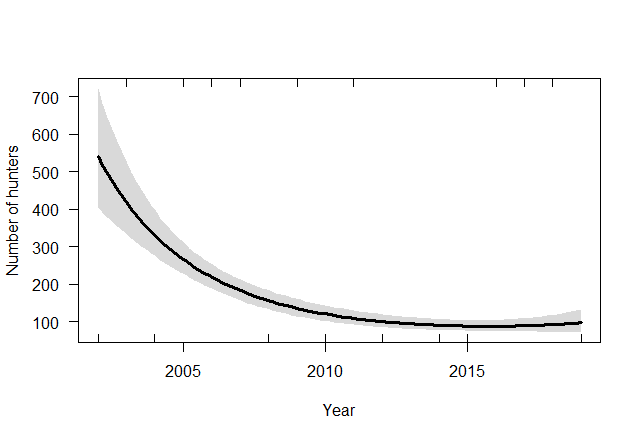
**

**Supplementary Fig. S1.** Relation between number of hunters accredited in the *Programa Curiyú* and years for 2002–2019 period. The number of hunters declines through the years. The negative binomial regression is shown (GLM χ: -0.57 (-0.69, -0.45), P < 0.0001, χ^2^: 0.29 (0.15, 0.43), P < 0.0001, Deviance explained (%) = 85.8). Grey area indicates the 95% confidence interval.


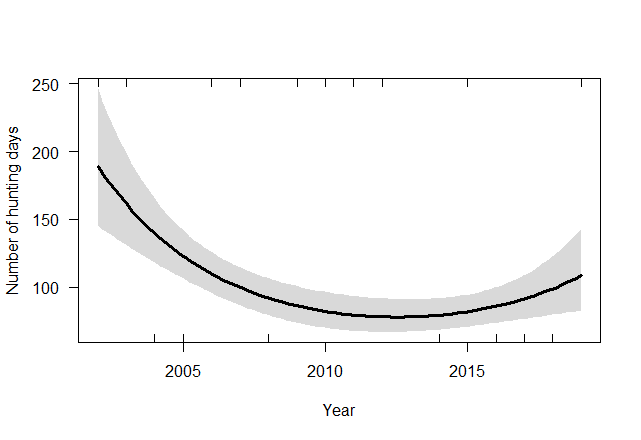


**Supplementary Fig. S2.** Relation between number of hunting days and years for 2002–2019 period. The number of hunting days declines through the years. The negative binomial regression is shown (GLM, χ: -0.19 (-0.3, -0.09), P = 0.0004, χ^2^: 0.24 (0.11, 0.36), P = 0.0003, Deviance explained (%) = 61.5). Grey area indicates the 95% confidence interval.


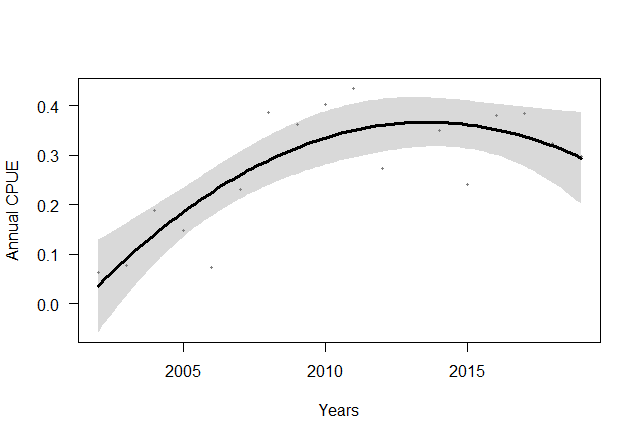


**Supplementary Fig. S3.** Relation between the annual capture effort (CPUE) and years for 2002–2019 period. The gaussian regression is shown (GLM, χ: 0.09 (0.05, 0.12), P = 0.0004, χ^2^: -0.07 (-0.12, -0.03), P = 0.005, Deviance explained (%) = 68.3). Grey area indicates the 95% confidence interval.


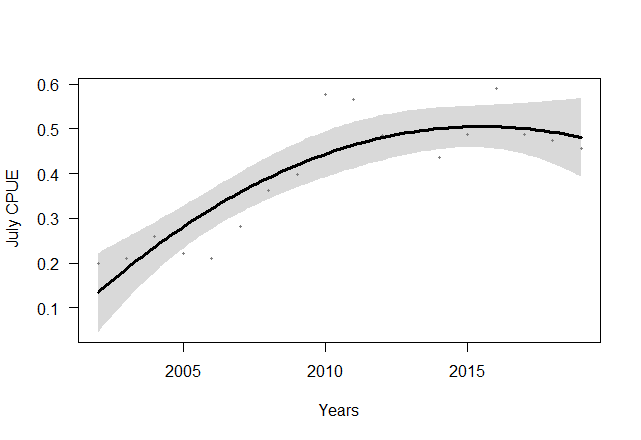


**Supplementary Fig. S4.** Relation between the capture effort (CPUE) in July and years for 2002–2019 period. The gaussian regression is shown (GLM, χ: 0.12 (0.08, 0.15), P < 0.0001, χ^2^: -0.06 (-0.1, -0.02), P = 0.01, Deviance explained (%) = 77.1). Grey area indicates the 95% confidence interval.


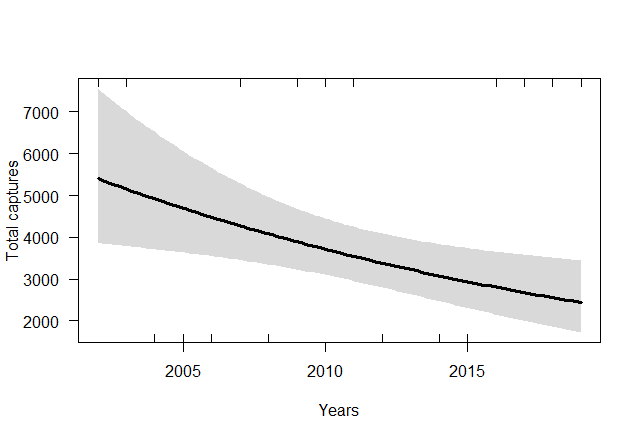


**Supplementary Fig. S5.** Relation between total captures of yellow anacondas (*Eunectes notaeus*) and years for 2002–2019 period. The negative binomial regression is shown (GLM -0.26 (-0.43, -0.08), P =0.007, Deviance explained (%) = 30.7). Grey area indicates the 95% confidence interval. The reduction in captures is related to the reduction in hunting effort and mean temperatures (see Table 3 in the main text).


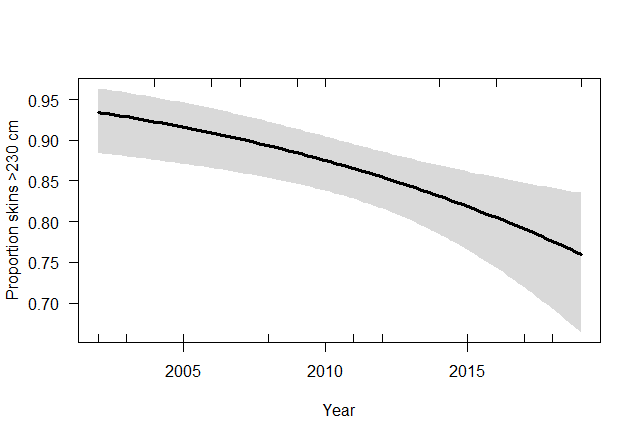


**Supplementary Fig. S6.** Relation between proportion of yellow anacondas (*Eunectes notaeus*) skins > 230 cm and years for 2002–2019 period. Over the years, the number of undersized individuals captured by hunters was reduced, positively contributing to sustainability and showing how hunter comply with the management system in place. The binomial regression is shown (GLM -0.48 (-0.79, -0.19), P = 0.001, Deviance explained (%) = 39.3). Grey area indicates the 95% confidence interval.


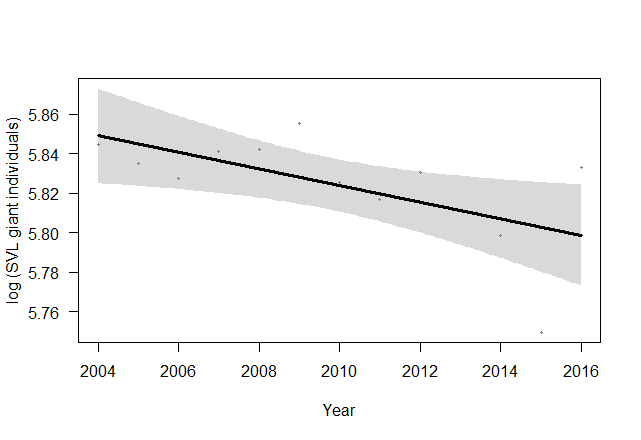


**Supplementary Fig. S7.** Relation between the mean SVL of yellow anacondas (*Eunectes notaeus*) giant skins (see text for definition) and years for 2004–2016 period. Giant sized individuals have slowly become rarer in the population, as it is typical of populations under management, but it was caused by extremely dry years. The gaussian regression is shown (GLM -0.02 (-0.03, -0.003), P = 0.04, Deviance explained (%) = 35.6). Grey area indicates the 95% confidence interval.


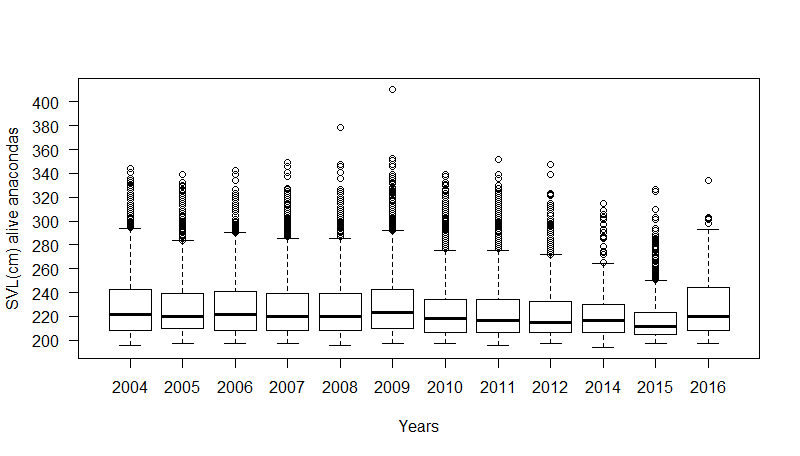
**Supplementary Fig. S8.** Average snout-vent length (SVL) of yellow anacondas (*Eunectes notaeus*) when harvested by the *Programa Curiyú* between 2004–2016 in northeastern Argentina. Declines typical of unsustainable practices are absent.

**
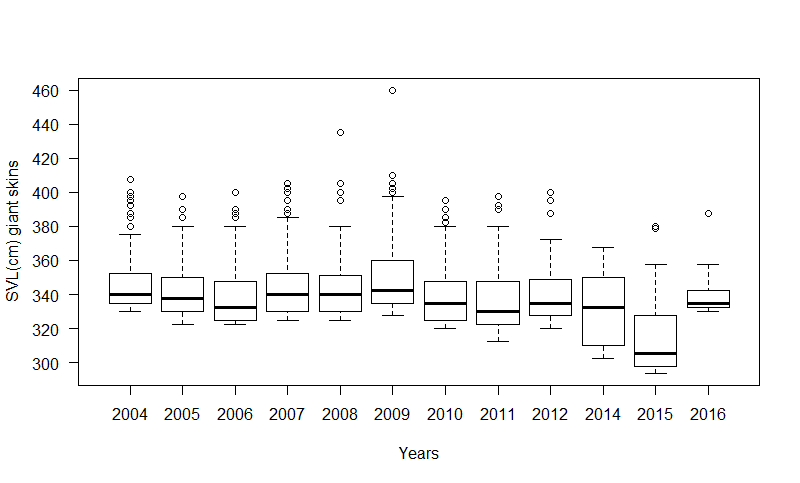
**

**Supplementary Fig. S9.** Estimated snout-vent length (SVL) of giant skins of yellow anacondas (*Eunectes notaeus*) (top 5% of the longest skins from each year) harvested by the *Programa Curiyú* in 2004-2016 in northeastern Argentina.

**Table S1.** GLM-based interaction models for proportion of captured females of yellow anaconda (*Eunectes notaeus*) between 2004-2019 in northeastern Argentina.

| **Explanatory variable** | **Coef.** | **P value** | **Deviance explained (%)** | **AIC** |
| --- | --- | --- | --- | --- |
| Mean temperature  Mean temperature^2^ | -0.006  -0.001 | 0.96  0.99 | 0 | 19.45 |
| Mean temperature | -0.006 | 0.96 | 0 | 18.57 |
| Year  Year^2^ | -0.07  -0.10 | 0.62  0.56 | 9.1 | 19.59 |
| Year | -0.12 | 0.32 | 6.7 | 18.9 |
| Hunting effort  Hunting effort^2^ | -0.28  -0.97 | 0.59  0.42 | 5 | 19.48 |
| Hunting effort | 0.03 | 0.91 | 0.1 | 18.63 |
