# Supplementary material for: Assessing the sustainability of yellow anaconda (Eunectes notaeus) harvest
Source: PLoS One. 2023 Jan 12;18(1):e0277629. doi: 10.1371/journal.pone.0277629 (PMC9836266; doi:10.1371/journal.pone.0277629)
Supplement: Attachment — Submitted filename: PLOS One review.docx [file pone.0277629.s002.docx]

**General Recommendations**

This manuscript does not address illegal harvest and the measures taken to prevent illegal sale of anaconda skins. This is a critical aspect of sustainable management of any game species. Are CITES tags required for sale? How is law enforcement involved? What are penalties for illegal harvest?

The hunting methods should be described in the Methods section. Are the hunting techniques selective? random? Can animals be released unharmed?

The annual size distribution of skins should be provided to determine the affects of hunting on the different size classes.

**Specific Recommendations**

Line 182	This equation should be explained. Why is the SVL + width multiplied by 10 and then divided by 2?

Line 187	Same here. What is magic about 11.71, 0.66, and 1.59? What are the significances of these numbers and how does this equation accurately represent the SVL of living snakes?

Line 256.	One sentence does not constitute a paragraph. This should be incorporated into another paragraph.

Line 264	Change “The current average of hunters is 182” to “The current average ***number*** of hunters is 182”

Line 277 Change “felled” to “fell”

Line 347 This statement with the 4 points should reference figures.

Line 414 – should this be a minimum SVL? Or total length of 230 cm?

Line 418 – this statement should have a figure reference.

Line 424 – are CITES tags utilized to track skins?

Line 300	Table 1 – the % of skins with SVL > 230 cm decline starting in 2011. What was the reason for this decline?

Figure 4 – The resolution is sufficiently low such that the y-axis labels cannot be read.
