# Supplementary material for: Assessing the sustainability of yellow anaconda (Eunectes notaeus) harvest
Source: PLoS One. 2023 Jan 12;18(1):e0277629. doi: 10.1371/journal.pone.0277629 (PMC9836266; doi:10.1371/journal.pone.0277629)
Supplement: Attachment — Submitted filename: Camera et al 2022 Response to Reviewers.docx [file pone.0277629.s003.docx]

PONE-D-22-12738

Assessing the sustainability of yellow anaconda (Eunectes notaeus) harvest

REQUIRED TOPICS TO ADDRESS

1. Edit for English language. The manuscript is well written but there are still many instances in which grammar or word choice is not correct. The authors should seek help from a colleague or professional editing service to ensure that the text is error-free.

**R**: We already expend high amounts of funds polishing the English of this manuscript, just to the corrections be ruined again during yet another round of revisions. We therefore would like to account on the Plos One team empathy to understand that, given the current economic crisis in Brazil, the manuscript should be sent to professional language review once we are sure it will be accepted. We cannot afford paying for multiple rounds of review anymore. The version you will receive will have its English edited by a native, and we hope this is the last time. We emphasize that English polishing was restricted to the main manuscript, while the cover letter and this response were not revised.

2. Data analysis needs to be clarified. As pointed out by the reviewers, it is sometimes difficult to follow what predictor variables were being analyzed and how model selection was being carried out. AIC-based model selection was described, but the actual selection itself was not shown in the manuscript.

**R**: That is right, we completely overhauled the AIC section so that it actually reflect the selection as presented in the table:

We used GLMs to assess the effects of the following predictive variables over the harvested population: mean temperature, hunting effort, mean temperature, and year. The explanatory variables were the parameters of the harvested population: total captures, mean SVL, skin SVL > 230 cm, and SVL of giant skins . During the pilot study the hunting of anacondas was extended outside the winter season, therefore, we exclude the first two years (2002–2003) from these analyses, specifically.

We standardized and centralized the explanatory variables to facilitate the interpretation of the estimated coefficients. Because year and hunting effort are highly correlated (r = -0.72), we built separate models to incorporate both variables. Among these, the best model was selected based on the lowest value of Akaike Selection Criteria (hereafter, AIC; 54). AIC estimates prediction error (54), and consequently can test the quality of different statistical models for different datasets (55). We performed all analyses in R software v.3.5.1 (56) and we used a significance level of 5%.

3. Justify the inclusion of the pilot study years or remove them. As pointed out by a reviewer, the first two years of data are from a pilot version of the wildlife management program and they may not reflect the general patterns in the rest of the dataset.

**R:** We will not strictly include or strictly exclude the data from pilot yeas, that would be wasteful. The data is compatible and comparable with everything else for some analysis, but not for others. On each occasion we took strict measures to afford data comparability. Each case is accompanied by a short explanation.

4. Reduce the strength of conclusions about sustainability. The data analysis in this paper is all based on harvest, which is not necessarily a direct reflection of population size and health, There are many examples from fisheries science in which harvest rates did not seem to be affected, but populations ultimately collapsed. There is a hint of a reduction in the size of females in the data presented, which may suggest that hunting is indeed having some sort of effect. The authors need to be more careful in how they present the "sustainability" of the program.

**R:** We did not analyze the variable “female size” because the data “skin’s size” and “sex” are highly correlated. Those data (skin’s size and sex) are taken in two different moments following the Programa Curiyú’s logistic data collection protocols. Thus, the argument pointed by the editor to diminish the power of our data comes from a not fully understood reading of the data showed in the MS.

Additionally, we would like to emphasize that whereas many of the issues pointed by the referee occur in managed populations, our data indicates a very clear trend in the reduction of the hunting effort each year. That reveals clear signs of sustainability, besides the fact that the population regulation seems to be performed by drought instead of hunting. Please mind that when you look again for the sightly size reduction: it is a product of severe drought, not hunting. Finally, the very long sampling period, combined with the fact that in this year—when the program completes 20 years—more than 6500 individuals have been harvested, adds robustness to our sustainability inference. Finally, “many examples” is not science, inference should not be made from idiosyncrasies, whereas the evidence in place on our case in quite clear.

5. Address comments from reviewers. The subject experts provided a lot of excellent feedback. The authors should carefully consider this in their revisions and response.

**R**: most reviewer’s appointment were carefully answered and correctly amended on the manuscript. In a few occasions were we disagree, rebuttals and justifications were given this document when we judged appropriated.

6. Data accessibility. The authors need to ensure that all data necessary to recreate their analyses is freely available.

**R**: Our data has been deposited in Mendeley repository, in the following link:

<https://data.mendeley.com/datasets/x8wwf3hzxb>

Please include the following items when submitting your revised manuscript:

A rebuttal letter that responds to each point raised by the academic editor and reviewer(s). You should upload this letter as a separate file labeled 'Response to Reviewers'.

A marked-up copy of your manuscript that highlights changes made to the original version. You should upload this as a separate file labeled 'Revised Manuscript with Track Changes'.

An unmarked version of your revised paper without tracked changes. You should upload this as a separate file labeled 'Manuscript'.

If you would like to make changes to your financial disclosure, please include your updated statement in your cover letter. Guidelines for resubmitting your figure files are available below the reviewer comments at the end of this letter.

PLOS ONE

Journal Requirements:

When submitting your revision, we need you to address these additional requirements.

1. Please ensure that your manuscript meets PLOS ONE's style requirements, including those for file naming. The PLOS ONE style templates can be found at

https://journals.plos.org/plosone/s/file?id=wjVg/PLOSOne_formatting_sample_main_body.pdf and

https://journals.plos.org/plosone/s/file?id=ba62/PLOSOne_formatting_sample_title_authors_affiliations.pdf.

2. Thank you for including your ethics statement: "N/A".

To comply with PLOS ONE submissions requirements, please provide the following information in the Methods section of the manuscript and in the “Ethics Statement” field of the submission form (via “Edit Submission”):

* Please indicate whether an animal research ethics committee prospectively approved this research or granted a formal waiver of ethics approval.* Please enter the name of your Institutional Animal Care and Use Committee (IACUC) or other relevant ethics board. Also include an approval number if one was obtained.

*	If anesthesia, euthanasia, or any kind of animal sacrifice is part of the study, please include briefly in your statement which substances and/or methods were applied.

For additional information about PLOS ONE submissions requirements for ethics oversight of animal work, please refer to http://journals.plos.org/plosone/s/submission-guidelines#loc-animal-research

Once you have amended this/these statement(s) in the Methods section of the manuscript, please add the same text to the “Ethics Statement” field of the submission form (via “Edit Submission”).

**R**: ethics statement has been substituted by a more detailed description of the ethical implications of our work.

3. Thank you for stating the following in the Acknowledgments Section of your manuscript:

[We thank the Ministerio de la Producción y Ambiente de la Provincia de Formosa, Fundación Biodiversidad and the Progama Curiyú, Office Vétérinaire Federal of Switzerland, Japan International Cooperation Agency, the Japan Wildlife Research Center, and all hunters for their logistical help. We thank the Conselho Nacional de Desenvolvimento Científico e Tecnológico (CNPq) for the financial support through Edital Universal number 003/2014 (process 155536/2014 and 456497/2014–5), and the research fellowship 3123038/2018-1. We thank Fundação de Amparo à Pesquisa do Estado de Mato Grosso (FAPEMAT) for grant conceded in Edital 017/2015.]

We note that you have provided funding information that is not currently declared in your Funding Statement. However, funding information should not appear in the Acknowledgments section or other areas of your manuscript. We will only publish funding information present in the Funding Statement section of the online submission form.

Please remove any funding-related text from the manuscript and let us know how you would like to update your Funding Statement. Currently, your Funding Statement reads as follows:

[The author(s) received no specific funding for this work.]

Please include your amended statements within your cover letter; we will change the online submission form on your behalf.

4. We note that you have indicated that data from this study are available upon request. PLOS only allows data to be available upon request if there are legal or ethical restrictions on sharing data publicly. For more information on unacceptable data access restrictions, please see http://journals.plos.org/plosone/s/data-availability#loc-unacceptable-data-access-restrictions.

**R**: as stated earlier, we included our data on Mendeley repository: https://data.mendeley.com/datasets/x8wwf3hzxb

In your revised cover letter, please address the following prompts:

a) If there are ethical or legal restrictions on sharing a de-identified data set, please explain them in detail (e.g., data contain potentially sensitive information, data are owned by a third-party organization, etc.) and who has imposed them (e.g., an ethics committee). Please also provide contact information for a data access committee, ethics committee, or other institutional body to which data requests may be sent.

b) If there are no restrictions, please upload the minimal anonymized data set necessary to replicate your study findings as either Supporting Information files or to a stable, public repository and provide us with the relevant URLs, DOIs, or accession numbers. For a list of acceptable repositories, please see <http://journals.plos.org/plosone/s/data-availability#loc-recommended-repositories>.

We will update your Data Availability statement on your behalf to reflect the information you provide.

5. We note that Figure 1 includes an image of a participant in the study.

As per the PLOS ONE policy (<http://journals.plos.org/plosone/s/submission-guidelines#loc-human-subjects-research>) on papers that include identifying, or potentially identifying, information, the individual(s) or parent(s)/guardian(s) must be informed of the terms of the PLOS open-access (CC-BY) license and provide specific permission for publication of these details under the terms of this license. Please download the Consent Form for Publication in a PLOS Journal (<http://journals.plos.org/plosone/s/file?id=8ce6/plos-consent-form-english.pdf>). The signed consent form should not be submitted with the manuscript, but should be securely filed in the individual's case notes. Please amend the methods section and ethics statement of the manuscript to explicitly state that the patient/participant has provided consent for publication: “The individual in this manuscript has given written informed consent (as outlined in PLOS consent form) to publish these case details”.

If you are unable to obtain consent from the subject of the photograph, you will need to remove the figure and any other textual identifying information or case descriptions for this individual.

**R**: we preferred to alter the photo to cover his face.

5. Review Comments to the Author

**Reviewer #1:**

1) The framework of this manuscript is evaluating the efficacy of an adaptive management program. The authors describe this as an experiment that learns from itself (I like that). However, I ask the authors to please provide more detail about this adaptive management program. What parameters are varied from year to year (presumably the number of permits given out and the length of the hunting season?). However, adaptive management makes its decisions based on observed results - so if the number of permits and the season length vary in response to something - what is it that they vary in response to? And could this changing management influence the results that the authors are looking for? I truly don't know and I think more details about the adaptive management program are required.

**R**: How explained in the line 393, there are two types of hunters working with the Programa Curiyú: the dedicated and the occasional. Thus, the number of permits results from the number of people that go to the PC to be credited and that is not under the PC control. However, as hunters numbers have been decreasing through years, and no signals of stress within the population was noticed, other parameters did not vary over time.

The variation in the hunting season results from the Programa Cuiyú staff experience. It was observed along the years that the climatic conditions were related to the hunting productivity. Despite of that, there was none research being performed about the topic until now. In this work we showed that the climatic conditions and PC productivity are related and how it works. We showed that when the mean winter temperature was higher the number of total captures decreased and that the increase in CPUE in the most productive month compared to the annual CPUE was significantly influenced by the lower temperatures. These data are tools for taking decision when to open the hunting season, especially having in mind that we all are experiencing times of global climatic changes. Thus, our MS is a very good example for other wildlife sustainable use in places around the world.

2) The authors include data from two pilot years. These years were extreme outliers in several regards with a hunting season that was about twice as long as all other years and significantly more hunters being given permits. I'm not entirely sure that these years should have been included in the analyses or perhaps analyses should be repeated with and without these years. I suspect that if they are removed many of the key results displayed in Fig 4 and highlighted in the Discussion will be different. How will this alter the conclusions?

**R**: As mentioned regarding an editor comment, we think both completely excluding or constantly including the pilot years would be wasteful, while in the occasions we used this data, the restrictions applied were in place to prevent misconceptions as the one noticed by the referee. In addition, in all cases where this data was used, there is a short explanation about how it was done.

3) Please provide rationale for the demographic parameters of interest earlier in the manuscript. I understand the difficulties in directly studying snake populations (its usually impossible) - thus you relied on these indirect metrics of population health. But because of this - I think more time and attention should be devoted to providing the reader rationale for why these parameters were of interest and how they directly relate to anaconda population viability. The authors describe some of this rationale in the 2nd paragraph of the Discussion but I think it needs to be featured in Introduction to better set up the rationale for the study design. In the Discussion the authors cite reference 47 evaluating harvest of reticulated pythons. The approaches between the two studies are extremely similar but the other study spends considerable time setting up the rationale that because snake abundance is difficult to measure, making assumptions that lack of observed changes to body size and demography of individuals suggests that harvest may be sustainable.

**R**: the following clarifications were added:

L84-87: This threshold guarantees that most individuals are able to breed, and allow anacondas to be managed from a effort perspective—instead of using quotas from an known population—since estimating snake populations through capture, mark and recapture is unfeasible [34].

L101-103: These demographic parameters where used given the cryptic nature of snake populations [38,39], which cannot be measure through other traditional ways like capture, mark and recapture [14].

4) I also think there are places where authors should temper their statements about showing the sustainability of this commercial harvest program and ensure they present their results in the context of these indirect measurements of population viability.

**R**: Despite we are using indirect measurements of population viability, a robust literature indicates that it is a powerful tool to suitability assessment. Every parameter analyzed says the management is sustainable. Thus, we are sure of our data and conclusions about the conservation power of the analyzed management program. Finally, we emphasize that this year the program reached 20 years of functioning and managed 6500 anacondas during the last hunting season.

5) L17: Population viability - not species viability

**R**: Word changed as suggested.

6) L22: Demographic parameters not biological

**R**: Word changed as suggested.

7) L23-24: I think this should be re-worded to be more cautious. This investigation does not directly evaluate population sustainability but rather uses indirect methods that hint at the maintenance of a healthy population.

**R**: We respectfully disagree with the referee. We have shown through this manuscript severe evidence that the management system in place has no effect over the managed population. The anaconda removal levels are within population recovery capabilities, and there are 20 years of evidence in favor of this sentence. There is plenty of data objectively tested regarding sustainability.

8) L35-36: Annual value of industry? Clarify

**R**: Word “annually” added for clarification.

9) L37: delete "nowadays"

**R**: Word deleted as suggested.

10) L49: delete the comma before "due"

**R**: Comma deleted as suggested.

11) L54-56: I don't understand the point being made here. Clarify. How do wildlife suffer when hunting is outlawed?

**R**: we edited the following sentences to improve clarity:

However—when outlawed—the continuous illegal use of wild meat, leather, or eggs means that traditional communities become marginalized by such activities [17]. At the same time, the wildlife suffer the consequences of irresponsible use, killing of undersized or immature individuals, which can lead to population crash [18].

12) L59: citation needed

**R**: The citation was added as suggested.

13) L67: replace specific with demographic

**R**: There are other parameters to have in mind when talking about wildlife management, but that would not be related to “demographic” parameters. For example, to caimans’ meat management, sanitary parameters need to be established by the managers. Thus, specific is the right word to be used here because we are paying attention that there are many other specific parameters to have in mind when managing a specific species. Thus, we argue that the word does not need to be replaced.

14) L76: "Have been". Please state the entity that manages Anaconda. Federal government?

**R**: Added as suggested.

15) L82: This tolerance might need to be clarified. What does this mean?

**R**: the following sentence was added in parenthesis, to improve clarity: (allowing estimates to be taken, instead of having an unknown rate).

16) L89: I think there should be a table or graph that shows how the adaptive management has changed the parameters of the harvest season in different years.

**R**: There were no changes in the harvesting parameters since its pilot phase. The pilot phase established the limit size for skins based on the species’ reproductive biology as explained in lines 127-130. Once that the number of credited hunters depends on the -number of people which request the accreditation, and that not reached so high, there was none changed this parameter. Here in this work we analyzed the climatic conditions and season duration and the data showed must be employed to maximize the productivity. Thus, despite the adaptive management framework allows for periodic changes in the management parameters, the Programa Curiyú never needed to do so.

17) Lines 90-102 - I think authors would benefit by telling the reader how they relate the 5 measurements they are taking to population sustainability. What are the predictions or how would a change in these values tell us about changing python populations. I believe this should be stated here.

**R**: We added the following explanatory paragraph:

The monitored parameters relate differently with the demographic cycles of a snake population. Capture rates can inform how hunting efforts vary annually, while they are expected to increase if the population is dwindling [42]. Body size positively correlates with fertility in female snakes [15,43], and information on their average and extreme sizes reveal the effects of both hunting and other factors (e.g. catastrophic droughts, predation or reduced prey abundance can reduce snake sizes; [44,45]). Proportion of undersized skins by its time allow learning the proportion of misdoing and how this is affected by other factors, such as increase in the required effort [7,39,46]. Together, these parameters allow us to understand better anaconda populations.

18) L96: What is rationale for this 230 cm cutoff?

**R**: We added the sentence “meaning a 230 cm dried skin.” Check the L83 to enlighten this question, but this is a size where most individuals have reached maturity.

19) L111: "as of the publication of this report"

**R**: Sentence changed as suggested.

20) L115: indices

**R**:Word changed as suggested.

21) L116: delete "managing"

**R**: Word deleted as suggested.

22) L126-129: re-write for clarity.

23) L130: delete "an individual of"

**R**: implemented exactly as suggested.

24) L155-160: Suggest providing more biological information about the anaconda that is relevant to population demographics. Age or size at maturity for each sex as well as reproductive capacity (how many clutches per year and how larger are clutches). Also important, when do they reproduce and does this correspond with hunting season?

**R**: We acknowledge the referee for this advice, the following sentence was added:

Females reach maturity within 2 m of SVL, and produce litters of 7-42 young every other year, and are normally maturing their eggs during the hunting season [34].

25) Equations 1 and 2: where are these derived from and how were they developed?

**R**: Equation 1 and 2—as pointed in the text—are products of reference 48 and were developed along the baselines of the management program. Readers keen to know the intricate details of snake leather drying will need to check this reference directly.

26) L247: how could a binomial distribution be used? These are continuous variables.

**R**: the text refer to the error distribution.

27) L270: rephrase. "established" doesn't' make sense

**R**: There is no “established” in the sentence, instead, “stabilized” is used to describe a plateau in our data on CPUE.

28) L285: "The average size of captured individuals"

**R**. Word changed as suggested.

29) L293-294: Is this a cause for concern?

**R**: At a perspective of the present discoveries, no. Here we are just describing the shape of our findings. The probable cause of this shape is discussed few times later in the MS in the section “Effects of hunting effort and environmental traits on the parameters of the harvested population”. At this moment we show that the mean SVL of giant skins, as well as the mean skin’s SVL > 230 cm were affected by mean winter temperature. In a future perspective, it decreases concerns as global temperatures will increase in the next few years.

30) Table 1: we are seeing a decrease in the percentage of "giant" snakes harvested - this is some cause for concern. Over last 3 years mean % is 75% where in previous years it was high 80 to 90%. Suggests fewer giants around. If larger anacondas have larger clutches, this could have population level effects and implications for recovery. Why the NAs for last 3 years?

**R**: There is a huge misunderstood with the showed data. There is no variation in “proportion” of giant snakes. This is a cut-off value we defined to call “giant snakes” and it is always 5%. The variation pointed by the reviewer is the % of skins larger than 230 cm, which does not reflect individual sizes.

31) Table 2: I do not understand what some of these statistics show.

**R**: We are not able to make changes in the table if the reviewer does not tell us what he did not understand.

32) L360-368 - This section gets very confusing regarding what the sample sizes in each study reference. That citation also highlights the need for decades long measurements.

**R**: the section was fully overhauled and now reads clearly:

A study analyzing the management of reticulated pythons (*Malayopython reticulatus*) in Indonesia argues that the minimum sample required for statistical detection of population changes would be analyzing 551 harvested individuals [58], an unlikely feat for most field studies. Additionally, the authors suggest that an annual decline of 5%, using samples sizes of 5,000 individuals, reveal population overexploitation. Declines greater than 10% over using smaller samples sizes—of about 1,000 individuals—also indicate overuse [58]. In our study—by means of a sample of 64,176 individuals—we conclude that the yellow anaconda harvest is sustainable, since the harvesting is not negatively regulating the analyzed parameters.

33) L368 - although you are seeing a decrease in the giants. I think this is important

**R**: We show that the average skins’ size is positively related to hunting effort and the hunting effort decreased over the period of study. Thus the average skins’ size is decreasing because the hunting effort is decreasing. The also show that the mean SVL of giant skins, as well as the mean skin’s SVL > 230 cm were affected by mean winter temperature, decreasing during the years when the mean temperature was higher. Thus, there is no signal of overhunting in our data, however, changes in the climatic condition (temperature) is a key factor influencing the size of harvested anacondas.

34) L375-381 - really good information that could be more effective if presented in the Introduction (or methods where the Anaconda is first described)

**R**: as per your former suggestions, information on that respect have also been included in the methods. However, given that is appears twice in the manuscript, a third mention in the introduction would be too much.

35) L379-380: good information in paragraph. But is there exploitation in surrounding regions or countries or is this program the only one? Important point.

**R**: Added information about absence of management programs in other countries.

36) L386: replace likely with May.

**R**: Word changed as suggested.

37) L387: describe methods more.

**R**: “manual capture” was added to clarify the method used to hunting anacondas.

38) L403: not sure I agree with this statement. Without inclusion of the pilot program - not sure that CPUE would vary across study.

R: We do not agree with the reviewer. Please, check the Fig. 4 C. The years 2005 and 2006 has lowers CPUE value. In fact, 2006 has the lowest CPUE value and in the subsequent years the CPUE increased abruptly until it reaches the higher value in 2011 and then stabilized. Unfortunately, you are square wrong.

39) L408-411: I don't know if this means that one male can't successfully inseminate a female

**R**: Actually multiple males are required. We rewrote the paragraph for clarity:

For snakes in general, long-term analysis of the sex ratio of a population is key for understanding the effects of harvesting programs [39]. Females represent a critical factor in managed populations. Harvest programs focused on non-reproductive males would in overall be more appropriate [58]. However, snakes of the genus *Eunectes* copulate in aggregations where one female is inseminated by several males. A reduction in the number of males increases the number of infertile eggs in females [15], since females approached by lesser amounts of males have more infertile eggs and insemination by multiple males is required. Thus, male harvest may have unexpected effects and unintended consequences on the reproductive biology of the species.

**Reviewer #2**

This manuscript is well-written and statistical methods seem sound. It would be nice if the authors would include more references to work conducted with the American alligator, which is unarguably the most successful sustained use conservation story in history.

**R**: we used mostly references of other developing nations with managements schemes more likely for Brazil, however we will include more on the American Alligator. Now we cite:

Eversole, C. B., Henke, S. E., Turner, B. L., Glasscock, S. N., Powell, R. L., Wester, D. B., & Ballard, B. M. (2018). A theoretical population and harvest model for American alligators (Alligator mississippiensis). Herpetological monographs, 32(1), 22-33.

Wilkinson, P. M., Rainwater, T. R., Woodward, A. R., Leone, E. H., & Carter, C. (2016). Determinate growth and reproductive lifespan in the American alligator (Alligator mississippiensis): evidence from long-term recaptures. Copeia, 104(4), 843-852.

Joanen, T., Merchant, M., Griffith, R., Linscombe, J., & Guidry, A. (2021). Evaluation of effects of harvest on alligator populations in Louisiana. The Journal of Wildlife Management, 85(4), 696-705.

**General Recommendations**

This manuscript does not address illegal harvest and the measures taken to prevent illegal sale of anaconda skins. This is a critical aspect of sustainable management of any game species. Are CITES tags required for sale? How is law enforcement involved? What are penalties for illegal harvest?

**R**: as per former and current referee comments, the following sections discuss these issues:

**L85-89**: However, a small harvest of undersized animals has also been tolerated. The proportion of undersized skins harvested can help to elucidate the levels of misconduct among accredited hunters, and to track if this willingness to bend established rules is being affected by factors such as the increase in required hunting effort.

**L115-119**: Proportion of undersized skins by its time allow learning the proportion of misdoing and how this is affected by other factors, such as increase in the required hunting effort [7,40,47]. Together, these parameters allow us to better understand anaconda populations, just as they do for other species under older and more well-established management initiatives, such as the American alligator [48].

**L454-464**: Finally, there is one possible problem for managed harvest we would like to discuss in detail: illegal harvesting. There is no poaching happening at a relevant scale. The program is the only initiative that manages the species, and skins available for international trade need to be labeled as legally derived from the managed population in Argentina. Historically, anaconda populations explored without the guide of a management program tended to produce small leathers, peaking around 160–180 cm [34], becoming easy to recognize as illegal and having a lower value for the buyer. Local indigenous populations in La Estrella rightfully hunt a few anacondas to produce trinkets and handicrafts for personal use, and the species is frequently killed when preying over domestic livestock [80], but none of these issues are impacting the demography of the species.

CITES tags are only for exportation, far away from La Estrella marshes.

The hunting methods should be described in the Methods section. Are the hunting techniques selective? random? Can animals be released unharmed?

**R**: Since anacondas are captured by hand (line 152 describes the very simple hunting method), hunting is selective and animals can be released unharmed. This sentence was added to the manuscript for clarity.

The annual size distribution of skins should be provided to determine the affects of hunting on the different size classes.

**Specific Recommendations**

Line 182	This equation should be explained. Why is the SVL + width multiplied by 10 and then divided by 2?

**R**: as mentioned earlier, details on the used equations can be found on published literature directly dealing with the adaptative management. As the manuscript is already long, and has clear objectives, the addition of further details on anaconda leather stretching properties seems quirky. We reorganized the citations, juxtaposing the references so that the reader can easily find where to study better in case of willing to learn these details.

Line 187	Same here. What is magic about 11.71, 0.66, and 1.59? What are the significances of these numbers and how does this equation accurately represent the SVL of living snakes?

**R**: these are related with gains and losses of length by anaconda skin, and are appropriately approached by specific literature that we cite. Further details of tannery will not be included here, and the reader can approach the literature in case he is willing to learn more. We are aware that most readers and the referee may be seen information over anaconda management for the first time, however it is unfeasible for a single paper to explain the complete management plan (just imagine someone putting the whole alligator management plan in a paper, including tannery details and felony consequences).

Line 256.	One sentence does not constitute a paragraph. This should be incorporated into another paragraph.

**R**: that was part of a table head and was successfully integrated to it. Thank you.

Line 264	Change “The current average of hunters is 182” to “The current average ***number*** of hunters is 182”

**R**: amended.

Line 277 Change “felled” to “fell”

**R**: amended.

Line 347 This statement with the 4 points should reference figures.

**R**: this whole section of the manuscript was so deeply overhauled that the comment doesn’t make sense anymore.

Line 414 – should this be a minimum SVL? Or total length of 230 cm?

**R**: minimum SVL, amended for clarity.

Line 418 – this statement should have a figure reference.

**R**: we added “(Fig. 4, C and D)” to the sentence end.

Line 424 – are CITES tags utilized to track skins?

**R**: yes.

Line 300	Table 1 – the % of skins with SVL > 230 cm decline starting in 2011. What was the reason for this decline?

**R**: decrease in the number of hunters.

Figure 4 – The resolution is sufficiently low such that the y-axis labels cannot be read.

**R**: that problem was caused by PLOS ONE merging system.
